# Transforming long-term post-acute care for the aging population through home infusion therapy in China: assurance for quality and safety of care delivery (Part 2)

**DOI:** 10.3389/fpubh.2026.1761870

**Published:** 2026-03-04

**Authors:** Wei Zuo, Zi Yin Zhou, Kayla Huynh, Dylan H. Do, Dan Mei, Tu Tran, Jianchun Yu, Hong Yang, Wei Chen, Bin Zhao, Vivian W. Y. Lee, Fan Zhang, Shaohong Wang, Shan Li, Austin-Phong Nguyen, Connie Vo, Huijie Shi, Jennifer Le

**Affiliations:** 1 Department of Pharmacy, Peking Union Medical College Hospital, Chinese Academy of Medical Sciences, Beijing, China; 2 Skaggs School of Pharmacy and Pharmaceutical Sciences, University of California San Diego, La Jolla, CA, United States; 3 College of Biological Sciences, University of California Davis, Davis, CA, United States; 4 Dartmouth College, Hanover, NH, United States; 5 Tylan Health, El Monte, CA, United States; 6 Huntington Hospital, Pasadena, CA, United States; 7 Department of General Surgery, Peking Union Medical College Hospital, Chinese Academy of Medical Sciences, Beijing, China; 8 Department of Gastroenterology, Peking Union Medical College Hospital, Chinese Academy of Medical Sciences, Beijing, China; 9 Department of Clinical Nutrition, Peking Union Medical College Hospital, Chinese Academy of Medical Sciences, Beijing, China; 10 Chinese University of Hong Kong, Hong Kong, China; 11 Department of Medicine Intensive Care Unit, Peking Union Medical College Hospital, Chinese Academy of Medical Sciences, Beijing, China; 12 Comfort Home Health & Hospice Care Inc, Long Beach, CA, United States; 13 Department of Pharmacy, Shenzhen People's Hospital, The Second Clinical Medical College, Jinan University, The First Affiliated Hospital, Southern University of Science of Technology, Shenzhen, China; 14 Center on International Pharmacy Education and Research, University of California San Diego, La Jolla, CA, United States

**Keywords:** aging population, healthcare costs, home health services, home infusion therapy, hospital burden, long-term post-acute care

## Abstract

**Background:**

China's healthcare system may benefit from the integration of home infusion therapy (HIT) combined with home health services (HHS) to address the urgent need for a sustainable long-term, post-acute care model for the aging population and those with chronic complex diseases. In this Part 2, we presented the current landscape of home- and community-based care models in China and formulated recommendations for successful implementation of HIT and HHS.

**Methods:**

We conducted a literature search up to October 2025 using MEDLINE, EMBASE, PubMed, Web of Science, and China National Knowledge Infrastructure.

**Results:**

To address the urgent need to support aging at home, China has initiated home- and community-based services nationwide. Despite these services, including a pilot of hospital-at-home, utilization remains low due to limited facilities, suboptimal service quality, and inadequate promotion. Notably, HIT and HHS that provide outpatient parenteral antibiotic therapy and total parenteral nutrition at home, and chemotherapy at infusion centers have not been adopted in China; yet this value-based modality has existed in western countries (including the United States, the United Kingdom, Australia, Canada, France, among others) for decades to provide patient-centric care that is safe and cost-effective. Implementing HIT requires a robust accreditation system, sterile compounding standards, technological integration, and professional training to ensure patient safety and quality of care.

**Conclusion:**

The shift of care from hospitals to homes through HIT supported by HHS holds great potential to alleviate healthcare costs, reduce hospital burden, and enhance patient comfort and dignity in China.

Part 1 is available at https://doi.org/10.3389/fpubh.2026.1761871.

## Background

Home infusion therapy (HIT) with home health services (HHS) offers a transformative and sustainable solution to address China's urgent need and demand for long-term, post-acute healthcare. In addition to relieving antimicrobial resistance and hospital burden, HIT and HHS provide value-based care for China's aging population and those with chronic complex medical conditions, improving overall healthcare affordability for both patients and the healthcare system. Part 1 presented the current need for HIT, particularly among the aging population, and assessed the structural design and cost-effectiveness of integrated HIT and HHS models. As a continuation in this part two, we presented the current home-based care models existing in China. We also formulated recommendations for successful implementation of HIT and HHS, incorporating cost-saving information of specific services [including outpatient parenteral antibiotic therapy (OPAT) and home total parenteral nutrition (TPN)], sterile compounding, technological innovation, and accreditation and certification standards, into existing infrastructure in China. Notably, HIT requires intravenous or subcutaneous administration of medications that are currently accessible only in hospitals throughout China. Patients currently in China require hospitalization for extended treatment duration since access to intravenous medications are unavailable outside the hospital, contributing to hospital overcrowding and antimicrobial resistance in landscape of the aging population.

## Methods

Using online databases, including MEDLINE, EMBASE, PubMed, Web of Science, and China National Knowledge Infrastructure, we searched for original research and review articles published in English or Chinese from database inception to January 2026. To ensure comprehensive coverage of policy, regulatory, and training-related literature that were not indexed in academic databases, we also manually searched the official websites of relevant government agencies. The key words used were home infusion, home parenteral infusion, outpatient infusion, outpatient parenteral therapy, outpatient parenteral antibiotic therapy, injection at home, home health, home care, and infusion center.

## Results

With the older population in China where currently 18.7% of the population is over 60 years old accounting for over 260 million people, this demographic is projected to be the fastest-growing over the next 30 years, reaching a peak of 488 million by 2050 and accounting for 35.6% of the total population (1–3). Chronic diseases lead to higher disability rates among older adults (4–6). According to 2019 data, over 180 million seniors in China suffer from chronic conditions, which account for more than 80% of the country's disability-adjusted life years and represent the largest health burden (7).

A survey revealed that a significant proportion of older individuals in China have diverse care needs: 38.1% require home nursing services, 12.1% housekeeping support, 11.3% rehabilitation care, 10.6% psychological counseling, 10.3% health education, and 9.4% daily care services (8). Addressing their care needs is imperative Changes in family structure, rapid urbanization, and the growing number of “empty-nest” older population are undermining traditional family-based care models for older adults with chronic diseases (9). Additionally, many seniors are reluctant to enter institutional care, which is often costly and limited in capacity (10).

### Current infrastructure for home health services in China

To address challenges in senior healthcare delivery and support aging at home, the Chinese government has, since 2008, promoted the development of home- and community-based services (HCBS) nationwide—providing medical care, daily living assistance, and social support (11, 12). Xu et al. conducted a study using a nationally available public database to examine the association between HCBS utilization and self-rated health among older adults with chronic diseases in China (13). This study showed that HCBS positively impacted the self-rated health of chronically-ill older adults. Given their poor physical health, limited mobility, and social isolation, HCBS offer targeted support by delivering accessible, cost-effective care to enhance their physical health, promote mental wellbeing through social engagement and emotional support, and enable aging in place by assisting with daily living activities and reducing the need for institutional care.

The implementation of HCBS in China, particularly to foster utilization of daily care and social support services, remains low mainly due to limited number of facilities, suboptimal service quality, and inadequate promotion. In 2019, only a minuscule 10% of communities in China were equipped with these care services and facilities (14). Inadequate staff training and unregulated service delivery are additional barriers to ensure effectiveness and uptake of HCBS. Limited awareness and promotion of daily care and social support services further hinder their use.

Beijing introduced the “9064” older population care service model in its 2015 special plan for older population care facilities (15). Under this “9064” model, 90% of seniors are cared for at home with the support of family members and socialized services, 6% of seniors receive government-funded community care services while residing at home, and the remaining 4% of elders reside in institutional care facilities (e.g., nursing homes and other long-term residence) for 24/7 care. In 2019, China's government issued the “medium- and long-term plan” for proactively addressing population aging (16). With medium-term goals through 2035 and long-term prospects extending to 2050, this plan emphasizes the development of high-quality older population care services that incorporate innovative models of care delivery for multiple chronic conditions; promotion of healthy living that includes resources and education on end-of-life preparations; and support for family and professional caregivers. The plan calls for the establishment of a multi-tiered older population care system that is home-based with the integration of medical services into communities and institutional care. Other policies, including China's tiered diagnosis and treatment policy with medical insurance reimbursements that steer patients toward primary care, and the gradual improvement of the urban primary healthcare system have contributed to the increase in community health centers or stations (14).

Hospital-at-home (HaH) is a care model that provides acute, hospital-level care to patients in their own homes for certain health conditions, including hypertension, diabetes, coronary heart disease, and hyperlipidemia. Similar to the USA's primary care model in treating chronic diseases generally without requiring hospitalization, China has adopted the HaH structure to alleviate hospital burden in a hospital-centric country with limited facilities and access to outpatient primary care services. An analysis of the HaH services at a community health center in New Pudong Area of Shanghai showed that most patients had hypertension (76%), diabetes (21%) and coronary heart disease (19%) (17). In 2023, Shenzhen—one of China's fastest-growing regions—established HaH services to provide care for 565,217 older residents aged 65 years and over (18). Managed by community health centers with hospital-linked online platforms, patients eligible for these services are those requiring intramuscular and subcutaneous medications, excluding any intravenous infusions.

Since the HaH model remains under pilot phase in China, the availability and access to these services across the country remains insufficient to meet population's urgent need and demand (19, 20). Furthermore, HaH is restricted to pre-hospitalization care for chronic conditions and does not serve those requiring long-term, post-hospitalization (i.e., post-acute) care needs as addressed by HIT and HHS. Currently, those requiring long-term, post-acute care require extended hospitalization, including those who would be treated with OPAT and home TPN in countries with HIT.

In China, the designation as HaH (or home) beds differ from hospitalization treatment beds to distinguish service capability. Accepted under current Chinese policies (e.g., management measures by local health authorities), this HaH model explored in Shanghai, Shenzhen, and other regions provides specific services such as oral medication, basic nursing care, rehabilitation guidance, and non-intravenous treatments in at home. Notably, according to current regulations in most regions, intravenous infusions (particularly antibiotics and chemotherapy drugs) are typically excluded from the routine services of HaH, making HIT distinct from HaH services. The Shanghai Municipal Health Commission issued the “*Standards for Home Hospital Bed Services in Shanghai*,” which states that high-risk procedures such as intravenous infusion, injections, complex wound care, suture removal, male catheterization, and gastric tube placement should not be undertaken at home for safety consideration (18). However, when these procedures are deemed necessary, authorized attending physician can perform such treatments after informed consent by patient (or family member) with full civil capacity. According to current regulations, high-risk injectable drugs are not permitted for routine home use due to safety concerns. As such, HIT with HHS that provide intravenous administration of antibiotics and parenteral nutrition at home and chemotherapy at infusion centers (which are outside of the hospital setting) has not been adopted in China; yet this value-based modality of care has existed in the USA for over 50 years to provide patient-centric care in a safe and cost-effective manner. These accumulated experiences in organizational structures, policies, management processes, insurance payment systems, and staffing models can provide a valuable reference for China as it adopts HIT, with appropriate modifications to align with China's infrastructure, to meet the demand to address older care.

In countries such as the United States, Canada, Switzerland, Poland, and others, home TPN is well-established and even a standard of practice in some countries to minimize hospital burden and prevent resistant hospital-acquired infections (21–26). Medicare data showed that a doubling in home TPN use from 39 per million residents in 1992 to 79 per million in 2013 (21). In USA, guidelines recommend home TPN for patients with intestinal dysfunction who are clinically stable and able to receive therapy outside an acute care setting (24, 25). A similar two- to three-fold increase was reported in Poland, with 53 per million citizens in 2020 receiving home TPN largely from malnutrition (22). Unlike Poland, home TPN for Canadian and Swiss residents was prescribed largely for underlying cancers (23, 27). According to a Canadian registry, indications for home TPN shifted between 2005–2008 and 2011–2014, marked by a rise in cancer patients (37.9 vs. 16.7%) while cases of short bowel syndrome declined (32 vs. 65.5%) (27). Despite significant utilization of home TPN in many countries, it has not been adopted in China, likely due to the need to develop policy, infrastructure, and professional and technical personnels to ensure its safe and cost-effective use. With China's increasing incidence of cancers coupled to the aging population, diversifying and expanding access to medications and healthcare services (e.g., access to anti-cancer drugs, telemedicine and artificial intelligence for remote consultation) especially outside of hospitals to decrease hospital burden and prevent multidrug resistant hospital-acquired infections, are encouraged in the near future (28). China accounts for approximately 24% of new cancer cases globally, with 30% of cancer-related deaths in 2020 (29).

### Evidence of cost-savings from international studies

Home infusion therapy is a specialized component of home healthcare. In particular, HIT involves the intravenous or subcutaneous administration of drugs or biologicals to an individual at an infusion center or home. The components needed to perform home infusion include the drug (e.g., antibiotics and immune globulin), equipment (e.g., a pump if needed), and supplies (e.g., tubing and catheters). Healthcare systems and payers are increasingly recognizing HIT's role in reducing hospital burden and overall costs. A recent review of six US-based studies on OPAT, enzyme replacement therapy, and continuous inotropic infusion reported that HIT resulted in substantial cost reductions compared with inpatient care. Savings ranged from $40,460 to $81,559 per patient (reaching as high as $120,500 for inotrope therapy), corresponding to $122 to $161.40 per day (30). Similarly, another systematic review consistently demonstrated that HIT was more cost-efficient, with average savings of $1,928 to $2,974 per treatment course (31).

Home infusion is a safe and recommended practice for patients, including the older population, receiving OPAT for infections requiring long-term treatment extending to several weeks to months in many western countries, especially the USA (32). A notable study showed an 85% cost reduction for OPAT ($122 per day at home vs. $798 per day in a hospital) (33). This translated into total cost avoidance of $646,000 to $834,000 per year. Another OPAT study reported a similar average of $130 per day (34). In addition, the US Office of Technology Assessment projected billions in savings if Medicare expanded coverage for HIT, primarily by reducing costs associated with hospital stays (35). Furthermore, a modeling study based on Medicare data estimated that nationwide OPAT implementation could save nearly $3 billion over 5 years (30).

In Hong Kong, You et al. (36) used a decision tree model to compare the direct costs of three treatment options for prosthetic joint infections caused by methicillin-resistant Gram-positive bacteria: inpatient vancomycin, vancomycin as OPAT, and outpatient oral linezolid. The clinical success was defined as resolution of baseline clinical presentation, with comparative estimates from published clinical studies. Monte Carlo simulations indicated that vancomycin OPAT was the most cost-effective, yielding cost savings of $2,313 vs. oral linezolid and $4,881 vs. inpatient vancomycin. From the perspective of Hong Kong's public health system, vancomycin OPAT offered the best value for cost-effectiveness.

In addition to OPAT, evidence demonstrates the cost-savings and support for the safe and effective use of home TPN as a form of HIT over hospital-based care. As a critical life-prolonging and potentially life-saving therapy for patients with severe gastrointestinal disorders due to various diseases including malignant cancer, home TPN should be administered to those patients unable to meet their nutritional requirements via the oral and/or enteral route, and who are clinically stable to be safely managed outside of the hospital. Studies have shown that home TPN is considerably less expensive than in-hospital PN (37). In fact, an international study reported the cost of home TPN as a percentage of hospital-based PN of 15%−70% (38). In France, the United Kingdom, and Canada, the financial burden of home TPN is almost entirely reimbursed by the National Health Service; in the USA, third-party carriers (including national insurance) pay 80% of the cost (39).

In Europe, the annual healthcare cost of home TPN for adult patients ranged from approximately €13,000 to €71,000, with the first year being more expensive than subsequent years (39–42). In fact, relative to the first year, costs decreased by 15% in the second year, 22% in the third year and 40% in the fifth year (39). Similar cost-savings for home TPN were reported in Latin American, with a cost reduction of 32% [mean difference –$1,498; 95% confidence interval (CI): –$1,203 to –$1,790 for a week of home vs. hospital TPN] (43). Overall direct costs were also lower by 36% over 1 month with home TPN (mean difference –$1,452; 95% CI: –$1,148 to –$1,756 36%).

In the USA, two studies have demonstrated that the average annual cost of home TPN was 24% up to a substantial 70% lower than hospital TPN (44, 45). Both of these studies estimated an average annual cost for home TPN was $10,072 to $19,700 per patient, compared to $42,987 to $73,720 per patient for hospital TPN. Another five studies compared the cost of home to hospital TPN also provided convincing evidence of the substantial cost savings of home TPN from 60 to 76% reduction (45–48). In Canada, the average cost of TPN during the final week of hospitalization was significantly higher than home TPN for the first month post-discharge ($567 vs. $405 per day; *p* < 0.0001), resulting in monthly cost savings of $4,860 per patient (95% CI: $2,700–$7,000) and even greater savings in older adults and those with cancer (49). In addition to studies demonstrating cost saving of home TPN, other studies have been conducted to assess its cost-effectiveness. Although home TPN not well promoted, the clinical and economic value of home TPN has been assessed in a randomized controlled trial to identify clinical applications and design national medical insurance policies within the Chinese healthcare system (NCT02066363) (50). The trial confirmed the clinical effectiveness of home TPN in patients with incurable gastrointestinal cancers. Notably, home TPN had an incremental cost-effectiveness ratio of $24,289.17, with an incremental cost of $2,051.18, and a quality-adjusted life year gain of 0.0844.

In Canada, a substantial savings of $19,232 per patient over 12 years, along with a 3.3-year increase in QALY, was reported with home TPN (51). This cost-savings was augmented with patient survival. A similar finding reported a saving of £170,306 per patient with home TPN compared to hospital TPN, demonstrating a 65% cost reduction (52). Cost-utility analyses indicate that the cost-effectiveness of home TPN improves with increased patient survival (53). This relationship is partly driven by the substantial expense of infectious complications, estimated at approximately US$10,000 per episode, often necessitating 10–15 days of hospitalization, and even more when catheter replacement is required (52, 53). Importantly, home TPN has been shown to reduce septic events by nearly fourfold compared with inpatient management, generating annual savings of around US$2 million in a single-center analysis (1).

### Economic evaluations in China

At present, large-scale, standardized service models of HIT have not been implemented in China. Consequently, publicly available and systematic economic evaluation studies are very limited. Furthermore, the structure of healthcare costs in China and the U.S. differs fundamentally. For instance, in the U.S., high hospitalization costs are driven by high drug pricing, technology cost, labor cost, and inflation outpacing reimbursement whereas in China, hospitalization costs (such as bed fees) are relatively lower and labor costs (e.g., nursing) are also comparatively modest. This, to some extent, limits the accuracy of directly extrapolating foreign data to the specific context of China. Nevertheless, based on inferences drawn from China's national conditions, we believe that there is potential for feasible cost savings in China.

First, China's national volume-based drug procurement policy has significantly reduced the prices of many drugs, which means that in HIT models, the cost advantage of the drugs themselves may be less pronounced than other countries. However, hospitalization costs in China—particularly bed fees, nursing fees, and certain examination costs—still account for a significant proportion compared to outpatient or home-based care. Therefore, under the premise of ensuring safety, transferring eligible patients to a home-based treatment environment could theoretically lead to direct savings in terms of hospital bed occupancy and related fixed costs.

Second, home-based treatment can reduce indirect costs for patients and their families, such as transportation, lost productivity, and caregiving expenses, which are particularly significant in a vast country like China. Finally, in China, where social health insurance is the primary payer with a current strong emphasis in controlling healthcare costs, any innovative model that can effectively reduce overall healthcare costs (particularly by hospital costs) may receive strong support from payers.

Notably, within China's healthcare system, drug pricing mechanisms (particularly influenced by national centralized procurement policies), and medical insurance reimbursement catalogs and payment methods differ fundamentally from Western models based on public and private insurance models. These structural differences imply that any cost-effectiveness analysis or model adaptation based on other countries' data must undergo rigorous scrutiny and restructuring within China's institutional and policy context.

### Sterile compounding for medication safety

To ensure patient safety from medication use, we underscore the importance to establish a framework for HIT pharmacy to prepare the intravenous medications under a strict sterile environment. The United States Pharmacopeia (USP) issues regulatory standards 797 for pharmaceutical compounding and 800 for hazardous drugs, both of which are used to supply HIT medications for use at infusion centers and the home (Table 1) (54, 55). In addition to providing the standards for compounding sterile preparation to ensure product sterility (i.e., free from microbial contamination, foreign chemicals, and other particulate matter), USP 797 addresses the stability of the compounded sterile preparations with appropriate beyond-use dates. Especially for home TPN use, both the sterility and stability of these complex admixtures are paramount, and the safe administration of home TPN requires a systematic approach from ordering to administration (54).

**Table 1:** Accreditation and certification programs for agencies and personnel in home infusion therapy and home health services in the United States.

**Program**	**Organization**	**Applicable to**	**Focus area**	**Condition or eligibility**	**Duration and renewal**
Home health agency accreditation	The Joint Commission (TJC)	Home health and home infusion agencies	Quality, safety, compliance with Medicare Conditions of Participation	Unannounced on-site inspections for each renewal period	Valid for 3 years (annual document reviews are required)
Home infusion therapy accreditation	Accreditation Commission for Health Care (ACHC)	Home infusion therapy providers and pharmacies	Standards for infusion pharmacy services, patient management, and quality assurance	Unannounced on-site inspections for each renewal period	Valid for 3 years (annual document reviews are required)
Home care accreditation	Community Health Accreditation Partner (CHAP)	Home health, hospice, and home infusion agencies	Organizational infrastructure, patient-centered care, clinical excellence	Unannounced on-site inspections for each renewal period	Valid for 3 years (annual document reviews are required)
Home infusion therapy accreditation	Centers for Medicare & Medicaid Services (CMS)	Medicare-enrolled infusion suppliers	Compliance with CMS standards for billing and care delivery	Unannounced on-site inspections for each renewal period	Valid for 3 years (annual document reviews are required)
USP 797 and 800	United States Pharmacopeia (USP)	Home infusion agencies, pharmacies, and healthcare facilities that compound sterile preparations or handle hazardous drugs	USP < 797>: establishes standards for sterile compounding to ensure quality, purity, and patient safety. USP < 800>: provides standards for safe handling of hazardous drugs to protect healthcare personnel, patients, and the environment	Compliance with USP < 797> and < 800> requirements for cleanroom design, environmental monitoring, aseptic technique, PPE, containment, decontamination, spill control, and documentation	Continuous compliance required; evaluated during accreditation, licensure, or regulatory inspections
**Personnel**					
Board certification in sterile compounding	Board of Pharmacy Specialties (BPS) (*proposed or evolving*)	Pharmacists	Advanced knowledge in sterile compounding for parenteral therapies	Passed the Sterile Compounding Pharmacy Specialist (BCSCP) certification examination	Valid for 7 years; continuing education and practice hours or re-exam required for renew
Home infusion therapy clinical competency certificate	Institution-Based or National Home Infusion Association (NHIA)	Pharmacist/nurse	Infusion device setup, medication preparation, aseptic technique, patient monitoring	Completion of structured in-house training and supervised practice	Institution-specific; often reviewed annually or biennially
Certified registered nurse infusion	Infusion Nurses Certification Corporation (INCC)	Registered nurses (RNs)	Clinical expertise in vascular access, infusion devices, pharmacology, and patient safety	Active RN license; 1,600 h infusion experience in 2 years	Valid for 3 years; continuing education and practice hours or re-exam required for renew
Certified home health aide (CHHA)	State Health Departments (U.S.)	Home health aides	Basic personal care, mobility support, vital sign monitoring	Complete accredited course and pass the certification examination	Continuing education and practice hours to maintain an effective state
Certified wound care nurse	Wound, Ostomy and Continence Nursing Certification Board (WOCNCB)	Nurses	Wound care management in home and post-acute settings	Licensed healthcare professional; requried clinical experience; complete accredited course and pass the certification examination	Valid for 5 years; continuing education or re-exam required for renew
Pharmacist certification in home infusion therapy (*custom/internal*)	Institution-based or via NHIA standards	Pharmacists in home infusion services	Aseptic preparation, drug stability, clinical monitoring, patient education	Complete accredited course and pass the certification examination	Valid for 7 years; continuing education or re-exam required for renew
Specialist in parenteral and enteral nutrition	National Board of Nutrition Support Certification (NBNSC)	RDs, RNs, MDs, PharmDs	Advanced competency in nutrition support, including TPN and EN	Licensed healthcare professional; 3 years of experience in nutrition support; pass the certification examination	Valid for 7 years; continuing education or re-exam required for renew
Certified nutrition support clinician	National Board of Nutrition Support Certification	Pharmacist/registered nurse/dietitian/physician	Parenteral and enteral nutrition therapy, monitoring, device management	Licensed healthcare professional; 2 years of experience in nutrition support	Valid for 5 years; continuing education or re-exam required
Basic life support (BLS)/advanced cardiovascular life support (ACLS)	American Heart Association (AHA)	All clinical staff	Emergency preparedness and life-saving skills for home settings	Complete accredited course and pass the certification examination	Valid for 2 years; continuing education or re-exam required for renew
Board certified sterile compounding pharmacist (BCSCP)	Board of Pharmacy Specialties	Pharmacist	Sterile compounding, USP < 797> and < 800> compliance, quality and safety	Active pharmacy license; practice experience and CE or postgraduate residency	Valid for 7 years; recertification via CE or exam
Basic life support (BLS)	American Heart Association	All clinical staff	Cardiopulmonary resuscitation (CPR), automated external defibrillator (AED), emergency response	Completion of accredited course	Valid for 2 years; renewal required
Advanced cardiovascular life support (ACLS)	American Heart Association	Registered nurse/advanced clinicians	Emergency cardiovascular care, advanced airway, pharmacologic interventions	Active clinical license; BLS certification	Valid for 2 years; refresher course or re-certification exam
Geriatric certified specialist (GCS)	American Board of Physical Therapy Specialties	Physical therapist	Geriatric mobility, fall risk management, functional rehab in older population	Licensed PT; 2,000 h in geriatrics or completio	
Wound care certified	National Alliance of Wound Care and Ostomy	Registered nurse/physical therapist/occupational therapist/physician	Wound assessment and treatment, debridement, dressing selection, infection prevention	Active license; completion of wound care training or CE program	Valid for 5 years; CE and renewal exam

The USP 800 standards focus on protecting healthcare workers and the environment from the risks associated with handling hazardous drugs, such as chemotherapeutic agents (55). Drugs that pose risks of carcinogenicity, teratogenicity, or reproductive toxicity even at low levels of exposure are required to be prepared using USP 800. Key provisions of USP 800 include facility and engineering controls, personal protective equipment, work practices, and disposal and waste management. Rigorous personnel training, cleaning and garbing procedures, and proper environmental controls, which contribute to the overall safety and quality of the final product, are addressed in both standards.

Both USP 797 and 800 represent the mandatory standards for sterile compounding practice in the USA. The complexity of complying with these USP standards underscores the necessity of a structured framework within the HIT pharmacy. Health information technology systems are invaluable in this endeavor, helping to manage workflows, track training competencies, maintain environmental monitoring logs, and ensure accurate beyond-use dating.

While adopting standards equivalent to USP 797 and 800 is paramount for patient safety, the significant capital investment required to build and maintain compliant cleanrooms presents a major financial barrier for widespread implementation in China's community health centers. We propose that a more feasible and scalable strategy for China is to leverage and extend existing high-capacity infrastructure, such as a “Hub-and-Spoke” model or centralized compounding centers. This can potentially reduce costs and optimize resource allocation. However, it also introduces challenges related to the stability and cost of drugs during long-distance transport, as well as issues of interest coordination and the legal chain of responsibility across institutions. Furthermore, in urban areas with substantial demand, a potential solution is establishing regional third-party sterile compounding centers dedicated to serving primary healthcare institutions and home care; this could serve as an effective complement to the hospital PIVAS model.

### Technological innovations

The innovative integration of HIT and HHS in China cannot be separated from the emerging technologies that enable infusion therapy beyond the hospital setting. Innovations in remote monitoring, portable infusion devices, smart home systems, and practical infusion safety tools have reshaped care delivery, allowing HIT and HHS to better serve China's aging population and improve access to long-term, cost-effective treatment after hospitalization, particularly in densely populated and underserved regions. A notable advancement is the integration of smart home ward system at Guangdong Second Provincial General Hospital (56). This system integrates telehealth, wearable monitors, and automated vital sign tracking to deliver hospital-level care at home for patient with chronic diseases including coronary heart disease, Alzheimer's disease, Parkinson's disease, and stroke, who are clinically stable and able to receive non-intravenous therapy at home. While artificial intelligence (AI) was mentioned, it remains unclear how it was integrated into care delivery but will play an important role in the future by analyzing real-time patient data to guide personalized treatment adjustments and predict potential complications. The AI application specific for HIT is emerging. Potentially, AI-driven predictive algorithms could analyze real-time infusion data (e.g., flow resistance, pressure patterns) to forecast potential catheter occlusions or pump failures, enabling preemptive intervention. Furthermore, through digital platforms, providers can maintain continuous communication with patients, remotely monitor, and promptly respond to potential complications (57, 58). This approach can potentially enhance HIT's capacity to reduce hospital stays, support early hospital discharge, and expand access to care while maintaining clinical safety.

China's national healthcare digitization strategy, including the “Internet + Health” initiative to expand the telemedicine infrastructure, can facilitate HIT and HHS implementation. Since 2019, integrating online care services into public health insurance has accelerated the adoption of virtual platforms for follow-up and chronic disease management (59). In 2020, the General Office of the National Health Commission released the Notice on Further Advancing the Pilot Program for “Internet + Nursing Services” (60). The six previously designated pilot provinces were instructed to continue their pilot work in accordance with this notice, while other provinces were required to designate at least one city, to pilot this program. The National Health Commission defined the basic requirements for service-providing institutions, personnel, responsibilities, and information technology. Furthermore, the deployment of high-speed 5G networks and mobile health applications can enable continuous remote monitoring of patients at home (e.g., early warning of sepsis risk in patients on home TPN, and personalized care adjustments). Providers can track treatment adherence, identify adverse events early, and adjust therapy remotely, thereby reducing the need for hospital visits and easing caregiver burdens. This telehealth infrastructure, potentially enhanced by artificial intelligence algorithms in the future for predictive analytics and personalized care adjustments, can strengthen patient safety and supports the scaling of HIT with HHS across China's diverse healthcare settings.

In addition to digital advancements, innovations in portable infusion technology have been critical for expanding HIT. Portable infusion pumps and implantable venous access devices have been used for cancer chemotherapy in Hong Kong, allowing patients to safely receive intravenous or subcutaneous medications without requiring hospitalization (61). In fact, this unique home service model allows for initiation of chemotherapy via multi-day continuous infusion by a nurse in the hospital, with continuation at home, and completion at the hospital for pump disconnection (62). These continuous infusion devices provide precise dosing and reliable rate control, enabling the administration of complex therapies such as OPAT, home TPN and palliative care outside hospitals (62–65). Interesting, refilling elastomeric infuser pumps for continuous infusions may further reduce device cost (66, 67). In addition, a portable infusion assistance device designed to manage multiple intravenous lines concurrently and prevent liquid infusion blockages was developed at Hebei Medical University (68). This device integrates fixed slots, three-way valves, and a mixing chamber to allow concurrent multiple infusion routes, bringing convenience to patients especially for long-term use. While studies have demonstrated cost savings with refillable elastomeric infusion pumps, these single-use portable devices are not currently covered by China's national or local basic medical insurance. To advance the development of HIT and ensure cost-savings, further studies are needed in China to assess home infusion equipment into the medical insurance payment framework.

Technological innovations within HIT and HHS can reduce reliance on inpatient infrastructure, lower healthcare costs, and optimize resource allocation. These benefits that align with China's growing emphasis on value-based care and efficient service delivery for the aging population and those requiring long-term, post-acute care for the complex medical conditions. In addition to new technology that enhances workflow, adequate training and certification of healthcare professionals are essential to ensure specialized knowledge and skills unique to HIT and HHS. Lack of standardized hands-on training and competency testing can increase the risk of medication errors, vascular access complications, infections, and adverse outcomes (68).

### Certification and accreditation

Accreditation and certification are essential for ensuring the safety, quality, and effectiveness of HIT and HHS (Table 1). These processes validate compliance with best practices and commitment to continuous improvement to ensure patient safety. As patients receive complex treatments such as OPAT, TPN, and biologics outside the hospital, it is critical that providers adhere to rigorous clinical standards and protocols. In the United States, accreditation of agencies by organizations like the Accreditation Commission for Health Care (ACHC), The Joint Commission (TJC), or the Community Health Accreditation Partner (CHAP) indicates that a HIT provider meets national standards of excellence for patient care, staff competency, infection control, and medication management (69–71). The ACHC offers accreditation with a focus on efficiency and pharmacy services; TJC is the US oldest and largest standards-setting and accrediting body in healthcare; and CHAP is the pioneer in community-based care with focus on HIT and home health services (69–71). These three independent, not-for-profit entities are CMS-approved accredited organizations for Medicare-certified HIT, home health and hospice services. They are responsible for ensuring compliance with Medicare's conditions of participation and promoting quality improvement in home and community-based care settings. Certification further validates that services are delivered by trained professionals using evidence-based practices, enhancing accountability, and reducing variability in care (72). These credentials improve clinical outcomes, reduce risks such as infections, build patient trust, and support compliance with regulatory and payer requirements (73, 74). Generally, China's regulatory focus lies in “institutional access approval,” and recognition of general professional qualifications (such as Registered Nurse and Licensed Pharmacist). There is a lack of specialized certification standards for both agency accreditation and personnel skill certification in the fields of HIT and/or HHS.

Accreditation and certification of HIT services and their personnel are tied to licensure and reimbursement. The home health value-based purchasing model rewards Medicare-certified home health agencies based on quality performance, using standardized outcome and process measures reported through the home health quality reporting program, such as the online assistance system for insurer submittals assessments and claims data. The HIT requirements mandate that providers delivering covered HIT services must be accredited and report on quality measures, ensuring safe administration and coordination with prescribing physicians (74, 75).

Comprehensive and targeted training specific to HIT is a cornerstone of safe and effective services, as it prepares both clinicians and patients to manage complex treatments in a non-clinical setting. The administration of HIT medications requires central lines or infusion pumps that necessitates specialized knowledge in sterile technique, line maintenance, dosing protocols, and complication management, all of which must adhere to accreditation standards and regulation. Nurses and pharmacists should be properly trained to assess the home environment, recognize ADEs, prevent infections, and effectively educate patients and caregivers on self-administration and monitoring. Ongoing, competency-based training improves patient outcomes, and builds confidence and trust in home-based care delivery.

### Challenges and assurance for HIT quality and safety in China

While the practice and experience of HIT from the United States and European countries have existed for decades, widespread and effective adoption of HIT in China will to face several intersecting obstacles—regulatory, reimbursement, technical, as well as socioeconomic and cultural constraints. Customizing a HIT model for China requires alignment with national policies that include regulatory and reimbursement structure, integration into Chinese clinical workflow and care delivery structures with proper accreditation and certification standards, and accommodation of China's HIT workforce reality.

The main challenge currently for HIT implementation in China stems from the lack of regulatory infrastructure for accreditation and certification to mitigate medical liability for providers and provide a risk management framework for patient safety. Firstly, the HIT agency and personnel must be appropriately accredited and certified, respectively (Table 1, see section Certification and Accreditation). In addition, professional liability (or malpractice) insurance protects healthcare professionals and businesses from claims of negligence, errors, or omissions that cause financial loss or harm to patients. Liability insurance is prudent regardless of practice in the hospital or HIT. Lastly, the shortage of technical professionals (especially nursing with proper training for HHS) presents another significant challenge for HIT implementation.

In consideration for building a tailored HIT model for China, the insurance policy and reimbursement structure must be addressed by key stakeholders, including payers (i.e., insurers, government, employers), providers (e.g., hospitals, physicians), and patients (i.e., policyholders). Leveraging existing policies effectively may expedite policy innovation. For example, the “Zero Markup” policy eliminates the old model of “using drug sales to subsidize medical services,” thereby creating a historic opportunity to build a sustainable HIT payment model centered on professional service value. Furthermore, the diagnosis-related groups/diagnosis-intervention packet model implements a “bundled prepayment” for hospitalization. To control costs and realize savings, hospitals are incentivized like HIT. Future efforts are required among key stakeholders to successfully integrate HIT into the payment framework.

There are socioeconomic and cultural considerations for HIT implementation. The advantage of HIT is that it improves the quality of life by enabling a patient to return home or work back to their daily routines. After the proper education and training by nurses, patients (or their family member) can self-administer certain treatments. Patients have access to a healthcare professional 24/7 who can instruct the patient to visit the hospital when deemed necessary for a significant adverse drug event or lack of clinical response to therapy. Notably, patients must visit physician clinics to ensure therapeutic response to therapy and nurses conduct regular home visits for direct monitoring. A challenge to be addressed is the shift of both patients and their families to acceptance and recognition of this new model. The training and education for both patients and HIT caregivers, coupled with the application of remote monitoring devices, are instrumental in facilitating HIT acceptance. Moreover, issues such as medical resource allocation and urban-rural disparities in China must be addressed in the design and future implementation of any HIT model.

**Figure 1:**
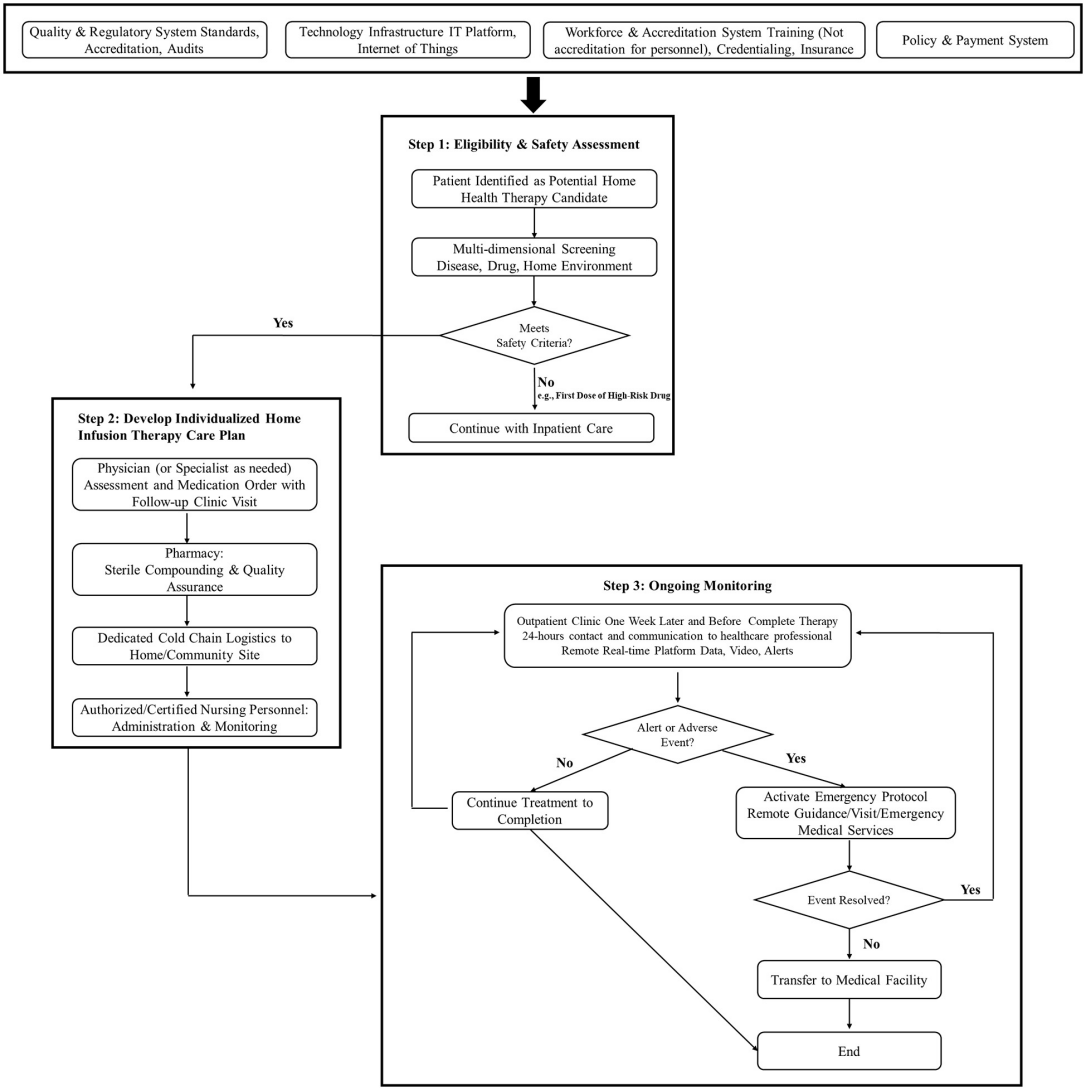
Continuum of care from hospital discharge to home infusion therapy.

We have presented the practices of HIT in different countries with decades of experience (Figure 1). However, realizing HIT potential in China will require a gradual, multi-year process of systemic development. Strategic, phased investment in regulatory alignment, workforce training, technological innovation, and the establishment of sustainable payment models will be essential. With such coordinated efforts, this value-based care model can evolve into a cornerstone of China's strategy to modernize care delivery and expand equitable healthcare access. Our narrative review has several limitations. One limitation was that the economic and operational evidence was largely from other countries' systems. As HIT currently exists only in other countries, only such published studies were included. Furthermore, fundamental differences in China's governance, pricing, insurance, and demographics require further evaluation to ensure applicability and feasibility of HIT in China, requiring multiple stakeholders to be involved to implement HIT.

## Conclusion

In China, shifting care from hospitals to homes through HIT supported by HHS holds significant potential to alleviate healthcare costs, reduce hospital burden, and enhance patient comfort and dignity. Comparable initiatives have not yet been developed or evaluated in China. Although international experiences cannot be directly applied in their totality, they offer valuable insights into program structure, implementation strategies, and potential outcomes. China's unique, rapidly evolving healthcare landscape with an aging population necessitates tailored HIT solutions that requires research to address unique systemic challenges.
